# Uniform Manifold Approximation and Projection (UMAP) Reveals Composite Patterns and Resolves Visualization Artifacts in Microbiome Data

**DOI:** 10.1128/mSystems.00691-21

**Published:** 2021-10-05

**Authors:** George Armstrong, Cameron Martino, Gibraan Rahman, Antonio Gonzalez, Yoshiki Vázquez-Baeza, Gal Mishne, Rob Knight

**Affiliations:** a Department of Pediatrics, School of Medicine, University of California, San Diego, California, USA; b Center for Microbiome Innovation, Jacobs School of Engineering, University of California San Diego, La Jolla, California, USA; c Bioinformatics and Systems Biology Program, University of California, San Diego, California, USA; d Halıcıoğlu Data Science Institute, University of California, San Diego, La Jolla, California, USA; e Department of Computer Science and Engineering, University of California, San Diego, La Jolla, California, USA; f Department of Bioengineering, University of California, San Diego, La Jolla, California, USA; Columbia University

**Keywords:** beta diversity, dimensionality reduction

## Abstract

Microbiome data are sparse and high dimensional, so effective visualization of these data requires dimensionality reduction. To date, the most commonly used method for dimensionality reduction in the microbiome is calculation of between-sample microbial differences (beta diversity), followed by principal-coordinate analysis (PCoA). Uniform Manifold Approximation and Projection (UMAP) is an alternative method that can reduce the dimensionality of beta diversity distance matrices. Here, we demonstrate the benefits and limitations of using UMAP for dimensionality reduction on microbiome data. Using real data, we demonstrate that UMAP can improve the representation of clusters, especially when the clusters are composed of multiple subgroups. Additionally, we show that UMAP provides improved correlation of biological variation along a gradient with a reduced number of coordinates of the resulting embedding. Finally, we provide parameter recommendations that emphasize the preservation of global geometry. We therefore conclude that UMAP should be routinely used as a complementary visualization method for microbiome beta diversity studies.

**IMPORTANCE** UMAP provides an additional method to visualize microbiome data. The method is extensible to any beta diversity metric used with PCoA, and our results demonstrate that UMAP can indeed improve visualization quality and correspondence with biological and technical variables of interest. The software to perform this analysis is available under an open-source license and can be obtained at https://github.com/knightlab-analyses/umap-microbiome-benchmarking; additionally, we have provided a QIIME 2 plugin for UMAP at https://github.com/biocore/q2-umap.

## OBSERVATION

An important step in microbiome research is visualizing the relationships between samples. In the study of microbial communities through next-generation sequencing (NGS), these comparisons are typically done through the visualization of beta diversities with principal-coordinate analysis (PCoA) (1) (see Fig. S1 in the supplemental material). Although alternatives such as conventional principal-component analysis (PCA), nonmetric multidimensional scaling (NMDS) (2), and t-distributed stochastic neighbor embedding (t-SNE) (3) are sometimes applied, PCoA in particular has been widely adopted by the microbiome community. Due to the high-dimensional and highly sparse nature of the data, which presents challenges on sequence count data (4, 5), one major benefit of PCoA over other methods on untransformed count data is that it accommodates a generalized distance matrix (of beta diversities, for the microbiome). This allows use of distance metrics that are better suited for sparse data (e.g., Bray-Curtis [6], Jaccard [7], and UniFrac [8]).

10.1128/mSystems.00691-21.2 FIG S1 Graphical abstract. UMAP can operate on distance matrices of arbitrary distance metrics (UniFrac, Bray-Curtis, Aitchison), similarly to PCoA. Download FIG S1, TIF file, 2.7 MB. Copyright © 2021 Armstrong et al. 2021 Armstrong et al. https://creativecommons.org/licenses/by/4.0/ This content is distributed under the terms of the Creative Commons Attribution 4.0 International license.

Uniform Manifold Approximation and Projection (UMAP) (9) is a method that has gained traction in single-cell genomics analysis (10). Whereas PCoA performs an eigendecomposition that focuses on linearly preserving the pairwise distances between the samples (global structure), UMAP uses a nonlinear graph construction and embedding method to optimize an objective that allows for a tradeoff between emphasizing local structures and preserving distances globally. This tradeoff is primarily controlled by the ‘n_neighbors’ and ‘min_dist’ parameters of UMAP. The ‘n_neighbors’ parameter controls the number of neighbors whose local topology is preserved, so global distances are preserved when it is high. The ‘min_dist’ parameter controls the minimum distance between samples in the embedding, which affects the spread of clusters. Low values of ‘min_dist’ allow UMAP to emphasize the similarity of dense clusters of samples, whereas larger values of ‘min_dist’ will focus on preserving distances more broadly.

Both UMAP and PCoA operate on a generalized distance (beta diversity) matrix, appropriate for microbiome data (Fig. S1). While the use of UMAP on microbiome data has been noted (11), the utility of UMAP on microbiome data remains underexplored. Using real data sets, we compared both visual qualities and quantitative measures of UMAP to those of PCoA on well-understood data sets. We additionally applied UMAP to data from the Human Microbiome Project (HMP) (12) to demonstrate its characteristics on a larger data set with more complex sources of variation.

**FIG 1:**
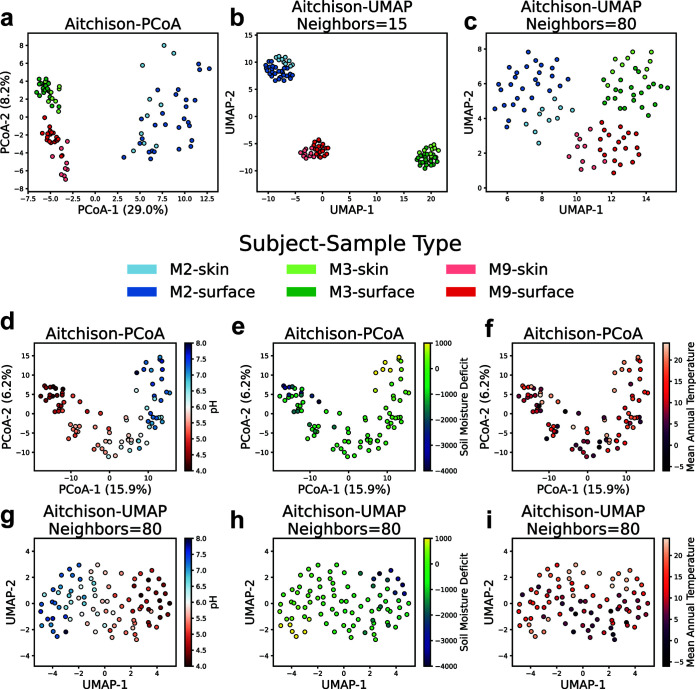
Comparison of PCoA and UMAP visualizations of cluster and gradient patterns on real data. The keyboard data set contains samples from three different subjects’ keyboards (surface) and their hands (skin). (a) PCoA on Aitchison distances (pseudocount = 1) demonstrates a strong separation between M2 and the other subjects, as well as separation between subjects M3 and M9. (b) A UMAP (n_neighbors = 15, min_dist = 1) visualization demonstrates stronger clustering by subject, with a different relative positioning of the clusters by subject. The plot also emphasizes clustering by sample type. (c) UMAP with an increased n_neighbors parameter (n_neighbors = 80, min_dist = 1) reflects the same relative positioning of clusters as PCoA. It also demonstrates the improved localization by sample type within subjects. (d) On the “88 soils” data, PCoA on the Aitchison distances demonstrates a horseshoe pattern with pH distributed along the horseshoe. (e) Soil moisture deficit is also distributed along the horseshoe, and (f) there is not a strong association between mean annual temperature and position on the PCoA. (g) In the UMAP (n_neighbors = 80, min_dist = 1), followed by centering/rotation with PCA, using the same distances, pH appears correlated with the first coordinate, (h) soil moisture deficit appears correlated with a sloped line across the pH gradient, and (i) there is a correlation between mean annual temperature and the second coordinate.

Discrete clusters are one common pattern that microbial communities can exhibit (13). The “keyboard data” from reference 14 contain 16S samples (99 samples, 1,399 features, 5% dense) from the keyboards and fingers of 3 subjects. PCoA on the Aitchison distances on these samples can recover the cluster structure of the subjects in the data (Fig. 1a). We compared this to UMAP (n_neighbors = 15 and n_neighbors = 80, min_dist = 1) and found that UMAP can also recover the cluster structure of the subjects (Fig. 1b and c). We also saw that UMAP produced two-dimensional coordinates with improved separation within subjects by sample type. To quantitatively assess the dimensionality reduction, we performed a supervised classification with linear discriminant analysis (LDA) as well as an unsupervised evaluation of clustering using the silhouette measure on the low-dimensional representations. The LDA classification, which solely measures separability, demonstrated higher accuracy of sample type (stratified by subject) on UMAP with two components compared to PCoA with two or three components for all subjects (Table S1). Silhouette scores (15), which measure cluster separation and density, demonstrated that host separation is improved with UMAP with a low ‘n_neighbors’ value, but not for a higher ‘n_neighbors’ value, which is likely due to the reduced distance between clusters in the UMAP coordinates with higher ‘n_neighbors’. The method with the highest within-host sample-type silhouette varied for each host. A simulated missing data analysis, where entries were randomly masked from samples, demonstrated that these results are sensitive to missing values (Fig. S3).

10.1128/mSystems.00691-21.4 FIG S3 Simulated missing data on keyboard study. A proportion of the entries of the table were randomly masked (20 repetitions per ablation level) from the feature table, and dimensionality reduction followed by LDA was run on each of the tables. Host accuracy is the accuracy for identifying the correct subject. The subject-type accuracies are specific sample-type accuracies specific to the individual. Download FIG S3, TIF file, 2.7 MB. Copyright © 2021 Armstrong et al. 2021 Armstrong et al. https://creativecommons.org/licenses/by/4.0/ This content is distributed under the terms of the Creative Commons Attribution 4.0 International license.

10.1128/mSystems.00691-21.5 TABLE S1 Linear discriminant analysis and cluster effect size on Aitchison embedding 10 different initializations for UMAP. Bold entries correspond to the maximum score for a problem. Download Table S1, XLSX file, 0.01 MB. Copyright © 2021 Armstrong et al. 2021 Armstrong et al. https://creativecommons.org/licenses/by/4.0/ This content is distributed under the terms of the Creative Commons Attribution 4.0 International license.

In dimensionality reduction, it is not only important for clusters to be separated; the positioning of clusters with respect to their similarity to other clusters, i.e., preserving global distances, is desirable. In the PCoA visualization (Fig. 1a), the samples of subjects M3 and M9 are similar to each other in the plot, and both are distant from M2. This corresponds with the expectation that M3 and M9 are more similar, because they shared an office. Additionally, this agrees with the original distances, where the mean Aitchison distance between M3 and M9 samples is 13.87 ± 0.11 (95% confidence interval [CI]), whereas the mean M2-M3 distance is 19.89 ± 0.11 (95% CI), and the mean M2-M9 distance is 18.94 ± 0.12 (95% CI). However, for UMAP with n_neighbors = 15 in Fig. 1b, the relative position of the clusters has changed (M9 is closer to M2 than it is to M3). Using the default ‘spectral’ initialization option, which is recommended for preserving global structure (16), we found that on only 34/50 initializations with different random seeds and n_neighbors = 15, UMAP produced clusters with the correct relative positioning. However, when we increase the parameter to n_neighbors = 80, which represents a large majority of the samples, the visualization retains separation by subject (Fig. 1c), and 50/50 initializations produced clusters with the correct relative positioning.

Ecological gradients are another common pattern that microbial communities can exhibit (13). The “88 soils” data from reference 17 contain 16S samples (88 samples, 5,628 features, 4% dense) from 88 different soils with additional measurements of the soil. A Bio-Env test (18) reveals that the top three soil variates corresponding with the Aitchison distances are pH, moisture deficit, and mean annual temperature (Table S2). In the PCoA of the Aitchison distances, which displays a horseshoe artifact (19, 20), pH is distributed along the horseshoe (Fig. 1d). To quantitatively assess the visualization of gradients in the data, similarly to reference 13, we calculated the Spearman correlation of the components of the ordination with the ecological variable. We found that soil pH is strongly correlated with the first component (Spearman *r* = 0.934) (Table S3). Soil moisture deficit is also distributed along the horseshoe (Fig. 1e), with PCoA-1 (Spearman *r* = 0.828). There is a mild correlation between mean annual temperature and the second PCoA coordinate (Spearman *r* = 0.313), although a pattern is difficult to see visually due to the horseshoe artifact (Fig. 1f).

10.1128/mSystems.00691-21.6 TABLE S2 Bio-Env-selected top 3 combinations of variables correlated with Aitchison distances. Download Table S2, XLSX file, 0.01 MB. Copyright © 2021 Armstrong et al. 2021 Armstrong et al. https://creativecommons.org/licenses/by/4.0/ This content is distributed under the terms of the Creative Commons Attribution 4.0 International license.

10.1128/mSystems.00691-21.7 TABLE S3 Spearman correlation of environmental variables with embedding. Download Table S3, XLSX file, 0.01 MB. Copyright © 2021 Armstrong et al. 2021 Armstrong et al. https://creativecommons.org/licenses/by/4.0/ This content is distributed under the terms of the Creative Commons Attribution 4.0 International license.

On the gradient problem, we fit UMAP with the parameters used with the keyboard data (min_dist = 1, n_neighbors = 80). Since the UMAP algorithm does not guarantee that the direction with the most variance in its output coordinates is axis aligned, we use PCA to identify the direction of maximum variance in the UMAP embedding and rotate the UMAP coordinates so that this direction is aligned with the *x* axis. The visualization shows reduced horseshoe-like warping, in contrast to the PCoA (Fig. 1g). Additionally, the pH gradient is highly correlated with the first principal component of the embedding (Spearman *r* = −0.931). Furthermore, the soil moisture deficit is displayed clearly across the diagonal of the embedding (Fig. 1h) and is correlated with both components of the axes (Table S3). Finally, the mean annual temperature has a much clearer association in two-dimensional UMAP coordinates compared to the first two components of PCoA, with a higher Spearman correlation with the second component (*r* = 0.478 for n_neighbors = 80, *r* = −0.604 for n_neighbors = 87). PCoA exhibits maximum Spearman correlation with mean annual temperature in its third component (*r* = −0.567). So, while a single axis of PCoA may be more correlated with the gradient, UMAP is able to display each of the gradients in fewer dimensions.

**FIG 2:**
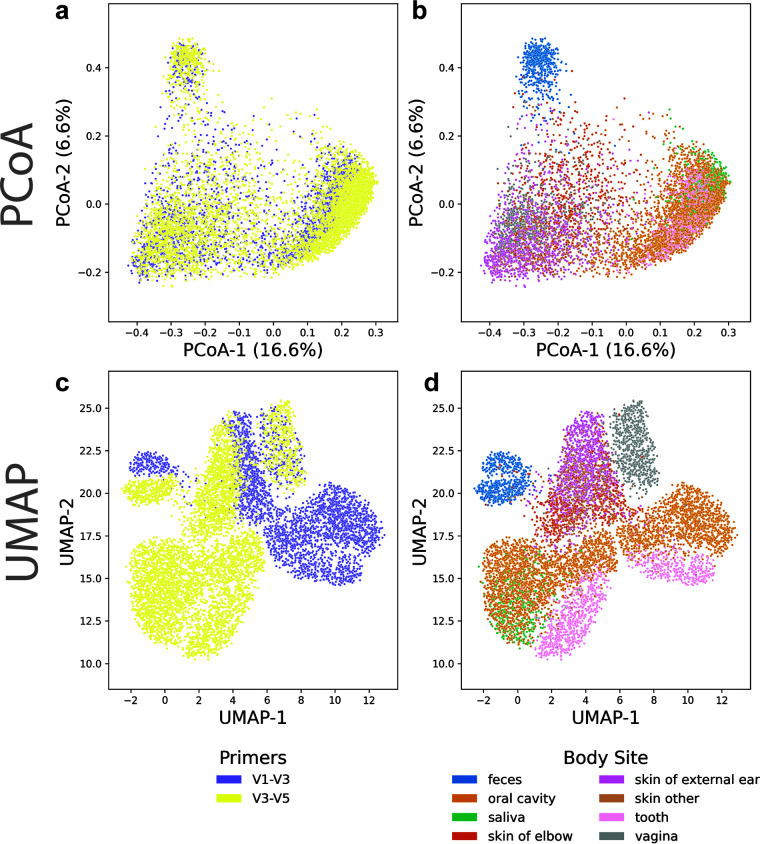
PCoA and UMAP comparison on 8,280 samples from the Human Microbiome Project (HMP). In the HMP data, when samples prepared with different primers are analyzed jointly, (a) there appears to be no separation between primers in the first two coordinates of PCoA and (b) mild separation by body site. In the same number of dimensions, UMAP is able to both (c) emphasize the differences between samples prepared with different variable regions and (d) improve clustering by body site. Both methods use the unweighted UniFrac distances on the HMP data rarefied to 1,000 sequences per sample.

Next, we compared PCoA and UMAP on data from the HMP (8,280 samples, 13,318 features, 0.08% dense) (12). These samples are from various body sites and individuals, with a large portion of samples processed with primers for two different variable regions of 16S. As noted in reference 21, the PCoA on unweighted UniFrac distances shows that differences in primers are not visible in the first two coordinates (Fig. 2a). Localization by body sites, however, is more apparent (Fig. 2b). Clustering by primer is instead visible in the third component of the PCoA (Fig. S2a), where clustering by body site is also apparent (Fig. S2b). We also fit a two-dimensional UMAP (min_dist = 1, n_neighbors = 800) to the same data. UMAP is able to separate a majority of the samples by variable region (Fig. 2c) and produces more distinct clusters by body site.

10.1128/mSystems.00691-21.3 FIG S2 Alternative views for PCoA and UMAP comparison on 8,280 samples from the Human Microbiome Project (HMP). (a) PCoA-3 shows separation by primers and (b) some symmetry of sample site by primer. (c) UMAP separates the primers as well as (d) body sites in only one dimension. Download FIG S2, TIF file, 2.6 MB. Copyright © 2021 Armstrong et al. 2021 Armstrong et al. https://creativecommons.org/licenses/by/4.0/ This content is distributed under the terms of the Creative Commons Attribution 4.0 International license.

To quantify the clustering in the HMP data, we trained a k-Nearest Neighbors (kNN) classifier on the respective variables with 10-fold cross validation and reported the mean accuracy on the test folds. We trained kNN models on the first one, two, and three components of the PCoA and fit UMAP embeddings for the respective number of dimensions. We found that kNN on a one-dimensional UMAP can outperform the sample site kNN for PCoA on up to 3 dimensions (Table S4). kNN trained on a two-dimensional UMAP was able to distinguish primers more accurately than kNN on the first two principal coordinates. This indicates that UMAP is capable of representing multiple sources of variability in microbiome data sets with thousands of samples more distinctly and in fewer dimensions than PCoA.

10.1128/mSystems.00691-21.8 TABLE S4 Comparison of 10-fold cross-validation accuracy of kNN in biological and technical variates. Entries for each target are ranked by mean accuracy. Download Table S4, XLSX file, 0.01 MB. Copyright © 2021 Armstrong et al. 2021 Armstrong et al. https://creativecommons.org/licenses/by/4.0/ This content is distributed under the terms of the Creative Commons Attribution 4.0 International license.

Finally, we explored a general-purpose recommendation for parameters. The parameters in this study were chosen to emphasize preserving the global structure of the data, by setting the ‘min_dist’ to its maximum of 1, increasing ‘n_neighbors’ from its default, and using default values for the rest of the parameters. In accordance with this goal, we set ‘n_neighbors’ to its maximum (*n* − 1 in general, 98 for soils, 87 for keyboard, and 8,279 for the HMP) and reran the previous analyses. With this parameter setting, the results remain largely unchanged (Table S4).

Our benchmarks demonstrate the potential for improved performance and interpretability for both cluster and gradient microbiome data by using UMAP with its parameters set with the intent to preserve global geometry. Given that the two algorithms provide different guarantees with respect to the preservation of distances in embeddings, we conclude that UMAP should be routinely used for microbiome analyses as a complement to PCoA. In order to facilitate using UMAP, we have made it conveniently available via QIIME2 (22) and Qiita (23) plugins.

10.1128/mSystems.00691-21.1 TEXT S1 Data processing and computational methods. Download Text S1, DOCX file, 0.01 MB. Copyright © 2021 Armstrong et al. 2021 Armstrong et al. https://creativecommons.org/licenses/by/4.0/ This content is distributed under the terms of the Creative Commons Attribution 4.0 International license.
