# Supplementary figures and images for: Uniform Manifold Approximation and Projection (UMAP) Reveals Composite Patterns and Resolves Visualization Artifacts in Microbiome Data
Source: mSystems. 2021 Oct 5;6(5):e00691-21. doi: 10.1128/mSystems.00691-21 (PMC8547469; doi:10.1128/mSystems.00691-21)

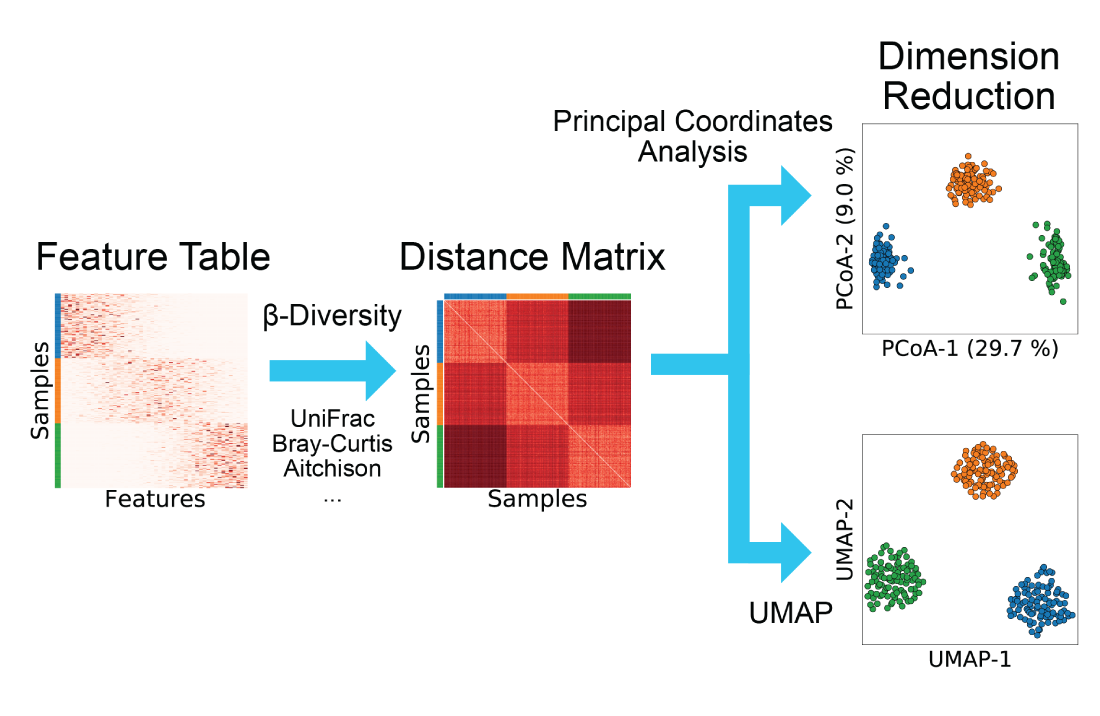

Supplement: FIG S1 [file msystems.00691-21-sf001.tif]

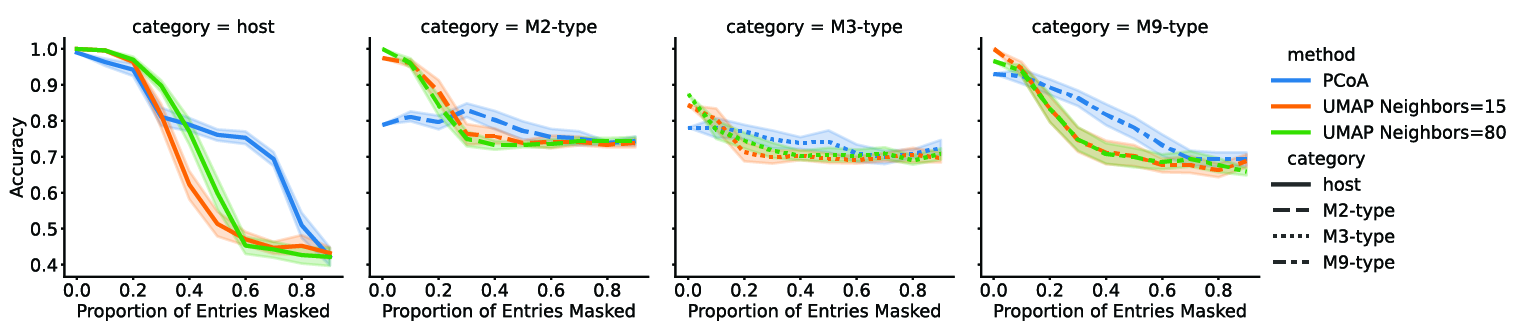

Supplement: FIG S3 [file msystems.00691-21-sf003.tif]

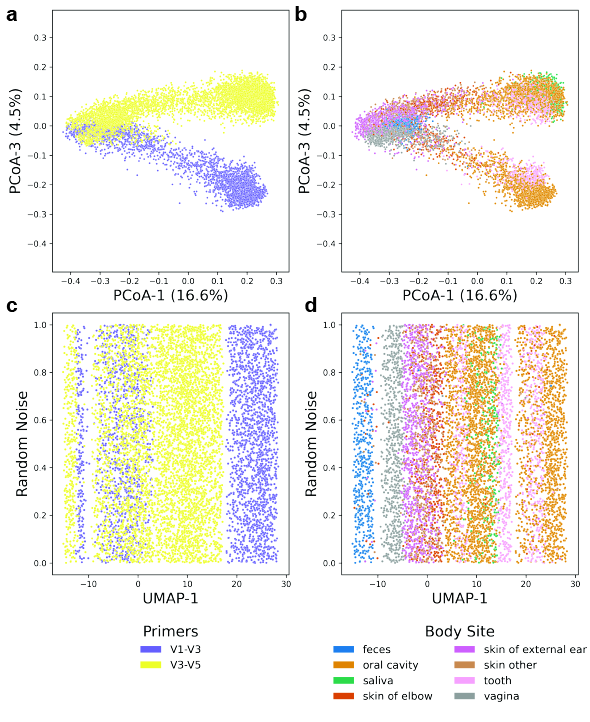

Supplement: FIG S2 [file msystems.00691-21-sf002.tif]
