# Supplementary material for: Uniform Manifold Approximation and Projection (UMAP) Reveals Composite Patterns and Resolves Visualization Artifacts in Microbiome Data
Source: mSystems. 2021 Oct 5;6(5):e00691-21. doi: 10.1128/mSystems.00691-21 (PMC8547469; doi:10.1128/mSystems.00691-21)
Supplement: TEXT S1 [file msystems.00691-21-s0001.docx]

### **UMAP reveals composite patterns and resolves visualization artifacts in microbiome data**

George Armstrong^1,2,3^, Cameron Martino^1,2,3^, Gibraan Rahman^1,3^, Antonio Gonzalez^1^, Yoshiki Vázquez-Baeza^2^, Gal Mishne^4,5^, Rob Knight^1,5,6,#^

^1^Department of Pediatrics, School of Medicine, University of California, San Diego, California, USA; ^2^Center for Microbiome Innovation, Jacobs School of Engineering, University of California San Diego, La Jolla, California, USA; ^3^Bioinformatics and Systems Biology Program, University of California, San Diego, California, USA; ^4^Halıcıoğlu Data Science Institute, University of California, San Diego, La Jolla, CA; ^5^Department of Computer Science and Engineering, University of California, San Diego, La Jolla, CA, USA; ^6^Department of Bioengineering, University of California, San Diego, La Jolla, CA, USA;

# Corresponding Author: robknight@ucsd.edu

### **Supplemental Material**

**Data Processing**

The keyboard (study id: 232), 88 soils (study id: 103), and Human Microbiome Project (study ids: 1927 and 1928) data were all acquired from Qiita. The keyboard (artifact id:v46809) and 88 soils (artifact id: 44763) data were both retrieved from Qiita with 90 NT trimming and closed reference picking. The human microbiome project data with both V1-V3 primers and V3-V5 primers were retrieved from Qiita with 100 NT trimming and closed reference OTU picking (artifact ids: 47414, 47420).

**Computational Methods**

PCoA's were computed with scikit-bio v0.5.6. UMAP was computed using umap-learn v0.5.1, using the default 'spectral' initialization. Aitchison distances were determined by taking the centered-log ratio of the raw sequence counts with a pseudocount of 1. UniFrac distances were computed with unifrac v0.20.2. All computation was performed on the Ubuntu 20.04 operating system.

Clusters were determined to be distorted (relative positions different than expected) if either M3 or M9 had a smaller distance to M2 than the distance between M3 and M9. Bio-Env was computed with scikit-bio v0.5.6, using "best subsets" selection. Linear Discriminant Analysis (LDA) was performed using scikit-learn v0.24.1. LDA was performed after simultaneously embedding all samples with UMAP. One model was trained to discriminate between each host. Subsequently, one model was trained per host to distinguish between surface and skin samples within the individual's samples. k-Nearest Neighbors was performed using scikit-learn v0.24.1 using 10-fold cross validation. Average test-fold accuracy values are reported.
